# Associations between maternal capabilities for care and nurturing care behaviours among mother-child dyads in Malawi and South Africa

**DOI:** 10.1371/journal.pgph.0005017

**Published:** 2025-09-02

**Authors:** Taryn J. Smith, Emmie Mbale, Michal R. Zieff, Chikondi Mchazime, Chloë A. Jacobs, Pious Makaka, Sadeeka Williams, Giulia Ghillia, Donna Herr, Marlie Miles, Thandeka Mazubane, Zayaan Goolam Nabi, Kirsten A. Donald, Melissa J. Gladstone

**Affiliations:** 1 Department of Women’s and Children’s Health, Institute of Life Course and Medical Sciences, University of Liverpool, Liverpool, United Kingdom; 2 Department of Paediatrics and Child Health, Kamuzu University of Health Sciences, Blantyre, Malawi; 3 Department of Paediatrics and Child Health, University of Cape Town, Cape Town, South Africa; 4 Neuroscience Institute, University of Cape Town, Cape Town, South Africa; Institute of Public Health Bengaluru, INDIA

## Abstract

Adequate nurturing care behaviours, including feeding practices, health-seeking and psychosocial stimulation, are crucial for the optimal health, growth and development of young children. However, several factors, recognised as maternal ‘capabilities for care’, can influence a mother’s capability to provide adequate care, namely knowledge/beliefs, physical health and nutritional status, mental health, autonomy, reasonable workload/time availability and social support. As part of the Khula birth cohort study, we aimed to investigate if maternal capabilities for care are associated with nurturing care behaviours among mother-child dyads in Malawi (n = 122) and South Africa (n = 206). When children were 10–16 months of age, mothers competed a series of self-reported sociodemographic, child diet and health and psychosocial questionnaires. Six maternal capabilities for care were considered: haemoglobin concentration, mental health, employment, decision-making autonomy, support for childcare and social support. The nurturing care behaviours were feeding practices, complete immunisation status appropriate to chid age and psychosocial stimulation within the home environment. Regression modelling assessed associations between maternal capabilities for care and each care behaviour, adjusting for child sex, maternal age and education level and household socioeconomic status. Associations between maternal capabilities for care and nurturing care behaviours differed by care behaviour and setting. Maternal employment and decision-making as measures of autonomy, support with childcare as a measure of reasonable workload and reported social support were the maternal capabilities most consistently associated with feeding practices, complete immunisation status and stimulation practices, although the direction of associations differed between settings. Maternal haemoglobin and mental health were associated with one care behaviour each (stimulation and feeding practices, respectively). Measuring and understanding how various maternal capabilities influence caregiving across contexts is essential for empowering caregivers to provide optimal care. Interventions, programmes and policies that seek to improve child health, growth and development through enhanced nurturing care practices should strengthen multiple maternal capabilities.

## Introduction

Adequate care is crucial for the optimal health, growth and development of children. Care refers to the provision of time, attention and support to meet the physical, mental and social needs of children and other household members [1]. Care behaviours include infant and young child feeding, health-seeking practices, psychosocial stimulation and hygiene practices. More recently, this has been conceptualised within the Nurturing Care Framework, encompassing five inter-related components for optimal child development: health, nutrition, early learning opportunities, responsive caregiving and safety and security [2]. The Nurturing Care Framework emphasises the need to invest in capacity building and empowerment of families, communities and service providers to nurture children who thrive.

Seminal conceptual frameworks on child nutrition and health have emphasised the importance of nurturing care for healthy growth and development [2–4]. Both the provision and quality of care can mitigate the adverse consequences of socioeconomic risks, including poverty [5]. However, recent evidence estimates that 25% of 3–4 year old children in low- and middle-income countries (LMICs) receive minimally adequate nurturing care (defined as access to at least one indicator out of two possible indicators in each of the five dimensions of nurturing care) [6]. The proportion of children receiving adequate nurturing care varies considerably across global regions, with those in sub-Saharan Africa estimated to have the lowest access to minimally adequate nurturing care (8%).

The family home represents the environment in which young children spend most of their time, being cared for by their primary caregivers, which in most contexts is the child’s mother. Thus, young children rely on their primary caregivers to provide nurturing care, and the resources available to caregivers could be critical in enabling their capabilities to provide nurturing care.

**Fig 1:**
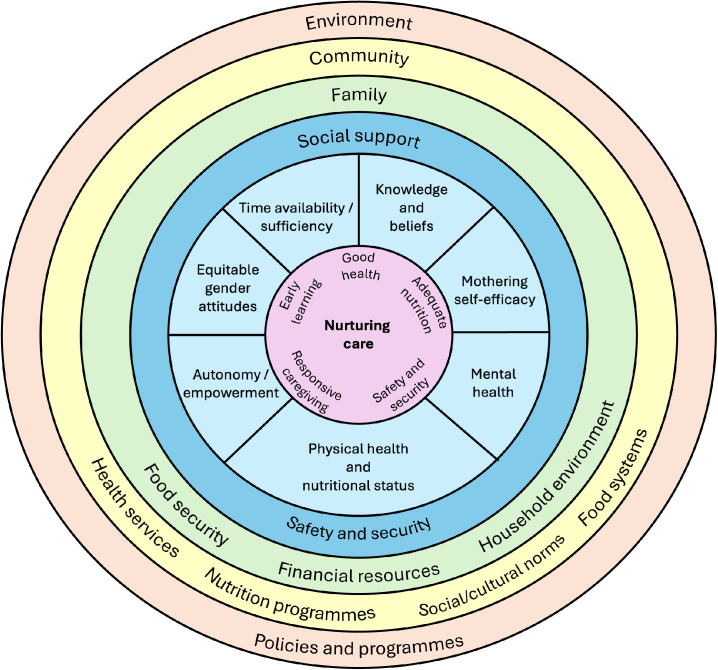
Multilevel factors influencing nurturing care within a social ecological model. Maternal capabilities for care (light blue) exist within family (green) and community (yellow) contexts and within enabling environmental systems (orange). Social support and safety and security (dark blue) can be measured at the individual-, family/household- and community-level. Adapted from Engle et al. (1999) [1] and Martin et al. (2024) [8].

‘Resources for care’ were first described by Engle and colleagues to define six intangible resources caregivers need to enact recommended nutrition and caregiving practices to provide nurturing care. These are: i) knowledge and beliefs, ii) physical health and nutritional status, iii) mental health, iv) decision-making autonomy, v) reasonable workload/time availability and vi) social support [1]. Further work has expanded this to describe ‘capabilities for care’ which are the skills and attributes of caregivers that determine their capability to translate resources (e.g., food, access to healthcare, education) into positive care behaviours that produce optimal health, nutrition and development outcomes for children [7]. The capabilities for care framework additionally included mothering self-efficacy and equitable gender attitude constructs. Individual-level maternal capabilities for care exist within family and community contexts (e.g., availability of and access to household and community resources) and within broader environmental systems (e.g., enabling policies and programmes such as parental leave policies and social welfare programmes) that enhance maternal capabilities (Fig 1).

There is considerable research investigating the relationship between a single maternal capability (e.g., mental health, social support) and certain care behaviours (e.g., child feeding practices) [8]. However, few studies have considered the full range of maternal capabilities for care and research is especially limited for maternal capabilities for care and psychosocial stimulation of children. In addition, findings are inconsistent across different contexts [9–11]. Failure to consider multiple maternal capabilities ignores the complexities and intricate challenges mothers can encounter in providing nurturing care to children in different contexts. Children have the right to develop to their fullest potential [12] and to fulfil this right it is imperative that mothers are prioritised and equipped with the resources and capabilities to provide nurturing care. Understanding the associations between multiple maternal capabilities for care and nurturing care behaviours may help identify areas wherein caregivers may benefit from additional support, as well as constraints among caregivers that may impair intervention impact. These insights could guide the design of interventions that more effectively support families in vulnerable contexts, empowering them to enhance their child’s nutrition, growth and development through nurturing care. Therefore, the aim of this study was to investigate if maternal capabilities for care are associated with nurturing care behaviours, specifically infant and young child feeding (IYCF) practices, health-seeking practices and early learning opportunities through psychosocial stimulation, among mother-child dyads in two different contexts in sub-Saharan Africa: Blantyre, Malawi and Cape Town, South Africa.

## Methods

### Ethics statement

Ethical approval was obtained from College of Medicine Research Ethics Committee at the Kamuzu University of Health Sciences, Malawi (Ref. P.11/21/3463) and the Human Research Ethics Committee at the University of Cape Town, South Africa (Ref. 666/2021). Written informed consent was obtained from one primary caregiver, usually the mother, after a detailed explanation of the study in a language appropriate to the family. Additional information regarding the ethical, cultural and scientific considerations specific to inclusivity in global research is included in the Supporting Information (S1 Checklist).

### Study design, setting and population

This is a secondary analysis of data collected from mother-child dyads who participated in a longitudinal birth cohort study, the Khula Study, in Blantyre, Malawi and Cape Town, South Africa. The primary aim of the study was to characterise brain development, specifically the structural and functional development of brain systems that underlie the emergence of executive functions over the first 1,000 days of life across diverse cultures and geographies. The study protocol has previously been described in detail [13]. Briefly, women >18 years of age who were pregnant (≥28 weeks’ gestation) or up to 3 months postpartum were eligible to participate. Women with multiple pregnancy, psychotropic drug use during pregnancy, infant congenital malformations and abnormalities (e.g., spina bifida, Down’s syndrome) and significant delivery complications (e.g., birth asphyxia) were excluded. A sample size of 300 participants at each site with longitudinal data from approximately 3–24 months of age was determined adequate power for the primary objective of the study [13]. Women were recruited from antenatal clinics in Blantyre district in southern Malawi (between 22 January 2022 and 6 August 2023) and Gugulethu in the Western Cape Province of South Africa (between 6 December 2021 and 29 November 2022). Blantyre is comprised of both urban and rural areas. Most homes are non-permanent small dwellings with improved drinking water sources. Around half of women have attended or completed primary education and around two thirds are employed. Communities are at high risk of malaria and undernutrition, particularly stunting among children <5 years of age and anaemia among children and women of reproductive age [13,14]. Gugulethu is a peri-urban suburb where housing comprises of formal dwellings or informal/shack dwellings. Most women have attended or completed secondary level of schooling. Communities are at a high risk of HIV, poor maternal mental wellbeing and exposure to violence [13,15]. Enrolled mother-child dyads in Blantyre and Gugulethu were invited for study visits at five time points between 3 and 24 months of age for a range of assessments including sociodemographic, child health, developmental, behavioural, neuroimaging, maternal psychosocial, child sleep and biospecimen sampling. This secondary analysis uses data collected at enrolment and the third study visit when children were approximately 10–16 months of age.

### Measures

#### Maternal capabilities for care.

Measures of maternal capabilities for care were guided by the UNICEF extended resources for care model [1] and the capabilities for care framework [7] and limited to data available within the Khula Study. This included nutritional status, mental wellbeing, autonomy, reasonable workload and social support. These measures were assessed at the third study visit when children were approximately 10–16 months of age, except haemoglobin concentrations that were measured at around 2–5 months postpartum. S1 Table provides detailed information on the definition, categorisation/scoring and timing of data collection for all maternal capabilities for care.

***Nutritional status:*** Haemoglobin concentrations were used as a measure of maternal nutritional status. A venous blood sample was collected from mothers postpartum for determination of haemoglobin by haematology analyser. The altitude of Blantyre is 1,039 meters above sea-level. Thus, as per the most recent World Health Organization (WHO) recommendations, haemoglobin concentrations in Malawi were adjusted for altitude by subtracting 8 g/L from the individual observed haemoglobin concentrations [16]. Gugulethu is at sea-level and hence no adjustments were made. Anaemia was defined as haemoglobin <120 g/L for nonpregnant women [16].

***Mental wellbeing:*** The Edinburgh Postnatal Depression Scale (EPDS) was used to assess maternal mental wellbeing. The EPDS is a 10-item brief tool to screen women for postnatal depression [17]. Mothers respond to statements about the frequency of depressive symptoms over the previous 7 days using a 4-point Likert scale (scored 0–3). Scores are obtained by totalling individual scores, with higher scores indicating a higher number and frequency of depressive symptoms (possible range 0–30). A score of ≥9 indicates a high risk of depression [18]. The EPDS has been validated in both Malawi [19] and South Africa [20].

***Autonomy:*** Maternal employment status (employed vs*.* not employed) was used as one measure of autonomy. Employment, particularly paid employment, is associated with greater levels of autonomy and employed women report greater participation in decision-making [21,22]. A second measure of autonomy, mother’s participation in household decision-making, was assessed using questions adapted from the Demographic and Health Survey [23]. This included mother’s involvement in household decisions about how mother’s own earnings are used, how partner’s earnings are used, mother’s healthcare, major household purchases and visiting family. A composite score was calculated by assigning a point for each response that indicated involvement of mothers in decision-making alone or jointly with a spouse (possible total score 0–5 in Malawi and 0–4 in South Africa).

***Reasonable workload:*** Support with childcare was used as a proxy measure for reasonable workload. A binary score for support with childcare (yes vs*.* no) was assigned based on whether mothers reported that they received support with childcare from a partner, grandparents, siblings, aunt, uncle or nanny/childminder.

***Social support:*** The Multidimensional Scale of Perceived Social Support (MSPSS) was used to measure social support. The MSPSS is a brief 12-item questionnaire to measure the respondent’s perception of the adequacy of support they receive from 3 different sources: a significant other, family, and friends [24]. The original version of the MSPSS includes 7 possible responses to each statement ranging from “strongly disagree” to “strongly agree” (scored 0–6). However, the MSPSS was adapted to the local contexts by reducing the number of response options on the Likert scale from 7 to 5 (scored 0–4; possible total score 0–48) [25]. A higher score indicates greater social support.

#### Nurturing care behaviours.

Nurturing care behaviours were guided by the Nurturing Care Framework [2] and limited to data available within the Khula Study. Three nurturing care behaviours were included: infant and young child feeding (IYCF) practices, health-seeking practices and psychosocial stimulation within the home, representing the nutrition, health and early learning components, respectively, of the Nurturing Care Framework. Maternal report on nurturing care behaviours was assessed at the third study visit when the children were approximately 10 – 16 months of age. S2 Table provides detailed information on the definition, categorisation/scoring and timing of data collection for all nurturing care behaviour indicators.

***IYCF practices:*** IYCF practices were based on mothers’ recall of foods and beverages her child consumed in the previous 24 hours using structured survey questions [26]. Continued breastfeeding was determined as the number of children who were receiving breastmilk at around 1 year of age (yes vs. no). Minimum dietary diversity (MDD) was calculated as the proportion of children who received food from ≥5 out of 8 defined food groups the previous day (yes vs. no). The 8 food groups were: i) breastmilk; ii) grains, roots and tubers; iii) pulses, nuts and seeds; iv) milk and dairy products; v) meat, poultry and fish; vi) eggs; vii) vitamin-A rich fruit and vegetables; viii) other fruit and vegetables. Minimum meal frequency (MMF) was the proportion of children receiving solid, semi-solid or soft foods at least the minimum number of times the previous day (3 times for breastfed and 4 times for non-breastfed children; yes vs. no). Minimum acceptable diet (MAD) was calculated as the proportion of children who consumed the MDD and the MMF in the previous 24 hours (yes vs. no).

***Health-seeking practices:*** Child immunisation status was used as a measure of health-seeking practices. A binary score for complete immunisations (yes vs*.* no) was assigned based on whether a child had received all essential vaccines appropriate for their age. Information on immunisations were taken from immunisation cards or, if not available, mother’s reporting.

***Psychosocial stimulation:*** The Family Care Indicators (FCI) was used to assess psychosocial stimulation and caregiver interaction within the home. The FCI is a brief tool developed by UNICEF that measures opportunities for stimulation and learning within the home environment of children <5 years of age [27]. Items are clustered into 4 subscales: i) availability of children’s books within the household (yes/no); ii) sources of play materials (score 0–3); iii) variety of play materials (score 0–7); iv) play activities with different caregivers in the previous 3 days (score 0–6). Scores were calculated for each subscale and a total FCI score as the sum for all 4 subscales (range 0–17), with a greater score indicating more stimulation and caregiver interaction with the child.

#### Covariates.

We pre-identified child, maternal and household factors known to influence child feeding practices, health-seeking practices and psychosocial stimulation (maternal age and education, household socioeconomic status) [28–32]. Child level covariates included child sex. Child recumbent length and weight were assessed when children were 10–16 months of age following standard protocols [33]. Age- and sex-specific z-scores were calculated using WHO growth standards [34]. Maternal level covariates included mother’s age and educational level at enrolment. Self-reported indicators of socioeconomic status (SES) included household size and composition, housing characteristics, access to utilities and household ownership of assets. A household SES score was calculated as the number of indicators available in the household from the following: i) electricity; ii) improved water source; iii) improved sanitation; iv) man-made flooring (Malawi) or type of dwelling (South Africa); v) improved cooking fuel (electricity/electric stove); vi) ownership of household assets. A score of 1 was assigned if the indicator was available in the household and 0 if not. Scores were summed for a total SES score (range 0–6). S3 Table provides detailed information on the definition, categorisation/scoring and timing of data collection for all covariates.

### Statistical analysis

Data were collected and managed using REDCap electronic data capture tools hosted at the University of Cape Town and subsequently analysed using SPSS version 29.0.1.0 (IBM SPSS Statistics, Armonk, NY). Descriptive statistics are presented as mean ± standard deviation or median (Q1, Q3) for continuous variables and frequencies (percentages) for categorical variables. Adjusted regression models were used to examine associations between maternal capabilities for care and nurturing care behaviours. Specifically, for the binary nurturing care behaviours (continued breastfeeding, MDD, MMF, MAD and immunisations), multivariable logistic regression models were used. For the continuous nurturing care behaviours (FCI total and subscale scores), associations were tested using linear regression. Separate models were run for each country. Each logistic and linear regression model included all six measures of maternal capabilities for care. For haemoglobin and EPDS scores, if there were significant associations based on continuous indicators, we repeated analyses with binary variables based on the cutoffs for each indicator described above. All logistic and linear regression models were adjusted for pre-identified child, maternal and household level covariates: child sex, maternal age, maternal education (none/primary/started secondary vs*.* completed secondary or above), and household SES score. Results are presented as adjusted odds ratio (OR) and 95% confidence interval (CI) for the logistic regression models and β and 95% CI for the linear regression models. All tests were two-sided at a 5% level of significance.

## Results

### Characteristics of mothers and their children

A total of 307 women in Malawi and 321 women in South Africa were enrolled and successfully contacted prior to the first study visit (when children were approximately 2–5 months of age) [13]. Of these, 122 and 206 mother-child dyads in Malawi and South Africa, respectively, had complete data for all maternal capabilities for care and nurturing care behaviours (assessed at the third study visit when children were 10–16 months of age) and were included in the analyses for health-seeking behaviours and psychosocial stimulation. Slightly fewer had complete data on IYCF practices (n = 121 Malawi; n = 184 South Africa). S1 Fig shows the full participant flow diagram.

Maternal, child and household characteristics are shown in Table 1. Mothers had a mean age of 27.2 ± 6.0 years in Malawi and 29.4 ± 5.7 years in South Africa. Children were 13.3 ± 1.6 months and 14.0 (12.8, 14.5) months in Malawi and South Africa, respectively. The majority (83.6%) of mothers in Malawi were married, while more women in South Africa were single/never married (64.1%). Almost all (97.6%) mothers in South Africa had some secondary education or above compared to 60.7% in Malawi. Household SES was slightly higher in Malawi and more households had access to improved drinking water sources and sanitation than in South Africa.

**Table 1:** Maternal, child and household characteristics^1^.

Characteristics	Malawi (n = 122)	South Africa (n = 206)
**Maternal characteristics**		
Age (yrs)	27.2 ± 6.0	29.4 ± 5.7
Marital status, n (%)		
Married	102 (83.6)	37 (18.0)
Cohabiting	–	36 (17.5)
Single	10 (8.2)	132 (64.1)
Divorced/separated	8 (6.6)	1 (0.5)
Widowed	2 (1.6)	0 (0.0)
Gravidity, n (%)		
1	23 (18.9)	36 (17.5)
2	39 (31.9)	70 (34.0)
3 – 6	47 (38.5)	100 (48.5)
Missing	13 (10.7)	0 (0.0)
Education, n (%)		
No formal education	7 (5.7)	0 (0.0)
Some primary education	24 (19.7)	0 (0.0)
Completed primary education	17 (13.9)	5 (2.4)
Some/started secondary education	32 (26.2)	101 (49.0)
Completed secondary education	32 (26.2)	75 (36.4)
Some/completed post-secondary education	10 (8.2)	25 (12.1)
**Maternal capabilities for care**		
Haemoglobin (g/L)^2^	118.3 ± 12.4	126.9 ± 10.6
Anaemia (Hb < 120 g/L), n (%)	57 (46.7)	45 (21.8)
EPDS score	2 (0, 6)	4 (0, 10)
EPDS ≥9	5 (4.1)	57 (27.7)
Occupation, n (%)		
Unemployed	9 (7.4)	109 (52.9)
Housewife	48 (39.3)	12 (5.8)
Farmer	4 (3.3)	–
Employed	54 (44.3)	73 (35.4)
Student	3 (2.4)	10 (4.9)
Other	4 (3.3)	2 (1.0)
Decision making score^3^	4 (1, 4)	4 (3, 4)
Support for childcare (yes), n (%)	85 (69.7)	147 (71.4)
MSPSS total score (0 – 48)	37 (35, 42)	35 (28, 40)
Significant other score (0 – 16)	13 (12, 16)	14 (12, 16)
Family score (0–16)	12 (12, 15)	12 (10, 15)
Friends score (0 – 16)	12 (11, 13)	10 (4, 12)
**Child characteristics**		
Age (mon)	13.3 ± 1.6	14.0 (12.8, 14.5)
Male, n (%)	66 (54.1)	108 (52.4)
Length-for-age z-score	-1.2 ± 1.2	-0.7 ± 1.2
Weight-for-age z-score	-0.3 ± 1.0	0.6 ± 1.3
Weight-for-length z-score	0.4 ± 1.2	1.2 ± 1.3
**Household characteristics**		
Household SES score^4^	5 (4, 5)	4 (3, 6)
Improved drinking water source, n (%)^5^	111 (91.0)	151 (73.3)
Improved sanitation, n (%)^6^	91 (74.6)	85 (41.3)
Household size	4 (4, 6)	4 (3, 6)
Number of children (0 – 17 yrs) in the household	2 (1, 3)	2 (1, 3)

EPDS, Edinburgh Postnatal Depression Scale; MSPSS, Multidimensional Scale of Perceived Social Support.

^1^Values represent mean ± SD or median (Q1, Q3) for continuous variables or n (%) for categorical variables.

^2^Haemoglobin concentrations in Malawi were adjusted for altitude [16].

^3^Maternal decision-making composite score developed by assigning a point for each response that indicated involvement of mother in decision making alone or along with a spouse with regards to decisions about how mother’s earnings are used, how partner’s earnings are used, mother’s healthcare, major household purchase and visiting family (total possible score of 5 in Malawi and 4 in South Africa).

^4^Household SES is the number of indicators available in the household from the following: electricity, improved water source, sanitation, man-made flooring (Malawi) or type of dwelling (South Africa), improved cooking fuel (electricity/electric stove), and ownership of household assets (range 0 – 6).

^5^Improved drinking water sources were defined as those protected from outside contamination [35].

^6^Improved sanitation facilities were defined as those that hygienically separate human waste from human contact [35].

### Maternal capabilities for care

Maternal haemoglobin concentration was lower among mothers in Malawi and a greater proportion of mothers had haemoglobin concentrations <120 g/L, indicative of anaemia (Malawi: 46.7%; South Africa: 21.8%) (Table 1). Mothers in South Africa had a higher median (Q1, Q3) EPDS score than those in Malawi (4 (0, 10) and 2 (0, 6), respectively). Additionally, 27.7% of South African mothers had an EPDS score ≥9, indicating a high risk of depression, compared to 4.1% in Malawi. Mothers in Malawi were largely employed (44.3%) or housewives (39.3%), while just over a third (35.4%) were employed and half (52.9%) reported being unemployed in South Africa. Mothers in both countries reported high levels of decision-making autonomy and approximately 70% reported receiving support with childcare in both countries. Levels of perceived social support were similar between the two countries, with slightly higher levels of support reported from a significant other than friends or family.

### Nurturing care behaviours

Continued breastfeeding at around 1 year was higher in Malawi than in South Africa (97.5% and 43.5%, respectively) (Table 2). In Malawi and South Africa, 53.7% and 45.1% of children, respectively, were fed a minimally diverse diet in the previous 24 hours. The prevalence of MMF was greater in Malawi than South Africa (74.4% and 65.8%, respectively), although in both countries less than half of children were fed a minimally acceptable diet (Malawi: 44.6%; South Africa: 34.2%). In Malawi, less than half (42.6%) of children had received all immunisations appropriate to their age, compared to over three quarters (77.2%) in South Africa. FCI total score, household books, and sources and varieties of play materials subscale scores were greater in South Africa, however mothers in Malawi reported more play activities with adults in the previous 3 days, particularly for telling stories, singing songs, taking the child outside and playing with the child (S4 Table).

**Table 2:** Prevalence of nurturing care behaviours^1^.

Nurturing care behaviours	Malawi		South Africa	
	n		n	
**IYCF practices**				
Continued breastfeeding, n (%)	121	118 (97.5)	184	80 (43.5)
Minimum dietary diversity, n (%)	121	65 (53.7)	184	83 (45.1)
Minimum meal frequency, n (%)	121	90 (74.4)	184	121 (65.8)
Minimum acceptable diet, n (%)	121	54 (44.6)	184	63 (34.2)
**Health-seeking practices**				
Full immunisation, n (%)^2^	122	52 (42.6)	206	159 (77.2)
**Psychosocial stimulation in the home**				
FCI total score (0 – 17)	122	6 (5, 8)	206	9 (7, 11)
Household children’s books (yes), n (%)	122	36 (29.5)	206	61 (29.6)
Sources of play materials (0 – 3)^3^	122	1 (0, 2)	206	2 (2, 3)
Varieties of play materials (0 – 7)^4^	122	1 (0, 2)	206	3 (2, 4)
Play activities (0 – 6)^5^	122	4 (4, 5)	206	3 (3, 4)

FCI, Family Care Indicators; IYCF, Infant and Young Child Feeding.

^1^Values represent median (Q1, Q3) for continuous variables or n (%) for categorical variables. Sample size for different assessments may vary.

^2^Child has received all essential vaccines appropriate for their age

^3^Homemade toys, shop bought toys, household objects

^4^Things/toys that play or make music, things for drawing or writing, picture books for children, things meant for stacking/constructing/building, things for moving around, toys for learning shapes and colours, things for pretending

^5^Read books or looked at picture books with child, told stories to child, sang songs to child, took child outside the home, played with child, counted or drew things with child

### Associations between maternal capabilities for care and nurturing care behaviours

#### IYCF practices.

In South Africa, mothers who were employed were less likely to continue breastfeeding their child at around 1 year compared to mothers who were not employed (p = 0.03) (Table 3). Mothers reporting higher levels of perceived social support were more likely to feed their child a minimally diverse diet (p = 0.05). Mothers who reported receiving support with childcare, as a proxy for reasonable workload, were less likely to meet the MDD (p = 0.05) and MAD (p = 0.04) recommendations compared to those who reported not receiving support with childcare. Surprisingly, mothers with higher EPDS scores (i.e., greater frequency of depressive symptoms) were more likely to feed their child a minimally diverse diet (p = 0.05) and a minimally acceptable diet (p = 0.04). However, this association was not significant when a binary EPDS variable (EPDS score ≥9 yes vs. no) was used. There were no significant associations between maternal capabilities for care and MFF in South Africa, or with any IYCF practices in Malawi.

**Table 3:** Associations between maternal capabilities for care and infant and young child feeding practices in adjusted analyses^1^.

Maternal capabilities for care	Malawi (n = 121)				South Africa (n = 184)			
	Continued breastfeeding	MDD	MMF	MAD	Continued breastfeeding	MDD	MMF	MAD
**Health and nutritional status**								
Haemoglobin	0.99 (0.86, 1.12)	1.01 (0.98, 1.04)	0.97 (0.93, 1.01)	1.01 (0.98, 1.04)	1.01 (0.98, 1.04)	1.02 (0.99, 1.05)	0.99 (0.96, 1.01)	1.03 (1.00, 1.06)
**Mental wellbeing**								
EPDS score	0.82 (0.53, 1.27)	0.95 (0.84, 1.08)	0.88 (0.75, 1.02)	0.89 (0.77, 1.02)	1.05 (0.99, 1.11)	1.07 (1.00, 1.13)*	1.03 (0.97, 1.10)	1.07 (1.00, 1.14)*
**Autonomy**								
Employed (yes)	0.86 (0.04, 2.09)	1.08 (0.47, 2.45)	2.13 (0.76, 5.95)	1.59 (0.70, 3.64)	0.46 (0.23, 0.93)*	0.49 (0.24, 1.02)	0.87 (0.43, 1.76)	0.65 (0.31, 1.39)
Decision making score	1.08 (0.55, 2.10)	0.96 (0.77, 1.21)	1.32 (0.99, 1.77)	1.00 (0.80, 1.25)	1.20 (0.95, 1.52)	1.11 (0.88, 1.41)	1.06 (0.84, 1.34)	1.09 (0.85, 1.40)
**Reasonable workload**								
Support with childcare (yes)	1.96 (0.20, 2.49)	1.05 (0.42, 2.67)	0.45 (0.12, 1.67)	1.21 (0.47, 3.10)	0.85 (0.41, 1.81)	0.46 (0.21, 1.01)*	0.84 (0.39, 1.80)	0.44 (0.20, 0.97)*
**Social support**								
MSPSS total score	1.06 (0.76, 1.52)	1.04 (0.97, 1.12)	0.96 (0.88, 1.04)	1.00 (0.93, 1.07)	1.01 (0.98, 1.05)	1.04 (1.00, 1.08)*	1.01 (0.98, 1.05)	1.04 (1.00, 1.08)
**Sociodemographic**								
Child sex (male)	1.00 (0.92, 1.09)	1.68 (0.76, 3.72)	0.48 (0.18, 1.30)	1.08 (0.48, 2.39)	0.81 (0.44, 1.51)	0.63 (0.33, 1.18)	0.84 (0.45, 1.57)	0.72 (0.37, 1.38)
Mother’s age	0.91 (0.68, 1.20)	1.00 (0.94, 1.07)	0.94 (0.87, 1.02)	0.99 (0.92, 1.06)	1.00 (0.94, 1.06)	1.05 (0.98, 1.11)	1.00 (0.94, 1.06)	1.04 (0.97, 1.10)
Mother completed secondary education or above (yes)	0.41 (0.02, 1.21)	1.35 (0.55, 3.34)	2.59 (0.77, 5.69)	1.33 (0.54, 3.28)	0.71 (0.37, 1.37)	1.69 (0.85, 3.35)	1.11 (0.56, 2.17)	1.21 (0.60, 2.46)
Household SES score	0.83 (0.20, 3.54)	1.41 (0.92, 2.16)	1.10 (0.71, 1.72)	1.45 (0.91, 2.29)	1.13 (0.89, 1.45)	1.38 (1.06, 1.80)*	1.22 (0.95, 1.56)	1.41 (1.07, 1.86)*

EPDS, Edinburgh Postnatal Depression Scale; MDD, minimum dietary diversity; MMF, minimum meal frequency; MAD, minimum acceptable diet; MSPSS, Multidimensional Scale of Perceived Social Support; SES, socioeconomic status.

Values are OR (95% CI).

^1^Adjusted for child sex, mother’s age, mother’s educational attainment (completed secondary education or above, yes vs. no), and household socioeconomic status score.

*p ≤ 0.05.

#### Health-seeking practices.

In Malawi, children of mothers who were employed were less likely to have received all their age-appropriate immunisations compared to children of mothers who were not employed (p = 0.04) (Table 4). There were no other statistically significant associations between maternal capabilities of care and child immunisation in Malawi or in South Africa.

**Table 4:** Associations between maternal capabilities for care and health-seeking practices in adjusted analyses^1^^.^

Maternal capabilities for care	Immunisations	
	Malawi (n = 122)	South Africa (n = 206)
**Health and nutritional status**		
Haemoglobin	0.99 (0.96, 1.03)	1.02 (0.99, 1.05)
**Mental wellbeing**		
EPDS score	1.09 (0.95, 1.24)	1.02 (0.95, 1.09)
**Autonomy**		
Employed (yes)	0.39 (0.17, 0.94)*	0.72 (0.35, 1.50)
Decision making score	0.92 (0.74, 1.16)	1.02 (0.79, 1.32)
**Reasonable workload**		
Support with childcare (yes)	0.73 (0.28, 1.92)	1.38 (0.61, 3.12)
**Social support**		
MSPSS total score	1.04 (0.97, 1.12)	0.97 (0.93, 1.01)
**Sociodemographic**		
Child sex (male)	1.85 (0.81, 4.21)	0.92 (0.46, 1.80)
Mother’s age	1.00 (0.93, 1.06)	1.00 (0.93, 1.06)
Mother completed secondary education or above (yes)	0.76 (0.31, 1.87)	0.98 (0.47, 2.04)
Household SES score	1.45 (0.93, 2.24)	0.77 (0.57, 1.00)

EPDS, Edinburgh Postnatal Depression Scale; MSPSS, Multidimensional Scale of Perceived Social Support; SES, socioeconomic status.

Values are OR (95% CI).

^1^Adjusted for child sex, mother’s age, mother’s educational attainment (completed secondary education or above, yes vs. no), and household socioeconomic status score.

*p ≤ 0.05.

#### Psychosocial stimulation.

In Malawi, maternal employment (p = 0.05) and higher levels of perceived social support (p = 0.02) were associated with lower levels of stimulation (i.e., lower FCI total score – less books, toys, play activities with caregivers) (Table 5). In South Africa, greater decision-making was associated with lower levels of stimulation (p = 0.005), but receiving support with childcare was associated with higher FCI total score, indicating more stimulation (i.e., more books, toys, play activities with caregivers; p = 0.001). There were no other statistically significant associations between maternal capabilities for care and FCI total score in either country.

**Table 5:** Associations between maternal capabilities for care and psychosocial stimulation within the home in adjusted analyses^1^.

Maternal capabilities for care	Malawi (n = 122)				South Africa (n = 206)			
	FCI total score	Sources of play materials	Varieties of play materials	Play activities	FCI total score	Sources of play materials	Varieties of play materials	Play activities
**Health and nutritional status**								
Haemoglobin	-0.09 (-0.04, 0.01)	-0.01 (-0.01, 0.01)	**-0.16** (-0.03, 0.00)*	0.04 (-0.01, 0.02)	0.03 (-0.04, 0.06)	-0.01 (-0.01, 0.01)	0.05 (-0.02, 0.03)	0.04 (-0.02, 0.03)
**Mental wellbeing**								
EPDS score	-0.08 (-0.17, 0.06)	-0.15 (-0.08, 0.002)	0.06 (-0.04, 0.08)	-0.07 (-0.08, 0.04)	-0.04 (-0.12, 0.07)	-0.12 (-0.05, 0.004)	-0.03 (-0.06, 0.04)	0.02 (-0.04, 0.05)
**Autonomy**								
Employed (yes)	**-0.18** (-1.43, 0.01)*	-0.01 (-0.29, 0.25)	-0.11 (-0.58, 0.14)	**-0.19** (-0.75, 0.00)*	0.002 (-1.06, 1.09)	-0.05 (-0.40, 0.18)	0.02 (-0.46, 0.62)	-0.02 (-0.52, 0.42)
Decision making score	0.13 (-0.06, 0.33)	0.05 (-0.05, 0.10)	**0.19** (0.01, 0.21)*	0.05 (-0.10, 0.11)	**-0.20** (-0.91, -0.16)**	**-0.25** (-0.29, -0.08)***	-0.12 (-0.35, 0.02)	**-0.16** (-0.35, -0.03)*
**Reasonable workload**								
Support with childcare (yes)	-0.004 (-0.84, 0.80)	**-0.47** (-1.12, -0.50)***	**0.46** (0.65, 1.47)***	-0.09 (-0.63, 0.23)	**0.24** (0.77, 3.09)**	**0.22** (0.16, 0.80)***	**0.20** (0.22, 1.39)**	**0.15** (0.01, 1.03)*
**Social support**								
MSPSS total score	**-0.23** (-0.14, -0.01)*	**-0.19** (-0.05, -0.003)*	-0.10 (-0.05, 0.01)	**-0.23** (-0.07, -0.01)*	0.03 (-0.05, 0.07)	-0.07 (-0.02, 0.01)	0.01 (-0.03, 0.03)	0.11 (-0.01, 0.05)
**Sociodemographic**								
Child sex (male)	**0.23** (0.19, 1.57)*	0.14 (-0.04, 0.48)	0.07 (-0.20, 0.49)	**0.18** (-0.002, 0.72)*	-0.08 (-1.58, 0.38)	-0.02 (-0.31, 0.22)	-0.12 (-0.94, 0.05)	-0.04 (-0.56, 0.29)
Mother’s age	0.07 (-0.04, 0.08)	0.08 (-0.01, 0.03)	0.01 (-0.03, 0.03)	0.09 (-0.02, 0.05)	0.00 (-0.09, 0.09)	0.09 (-0.01, 0.04)	-0.06 (-0.06, 0.03)	0.04 (-0.03, 0.05)
Mother completed secondary education or above (yes)	**0.23** (0.14, 1.72)*	0.09 (-0.15, 0.44)	0.13 (-0.11, 0.67)	0.15 (-0.10, 0.72)	0.02 (-0.94, 1.16)	-0.004 (-0.29, 0.28)	0.01 (-0.50, 0.55)	0.05 (-0.31, 0.61)
Household SES score	0.14 (-0.08, 0.63)	0.10 (-0.06, 0.21)	0.02 (-0.19, 0.20)	0.12 (-0.08, 0.30)	0.03 (-0.31, 0.46)	-0.07 (-0.15, 0.06)	0.07 (-0.10, 0.29)	-0.004 (-0.17, 0.16)

EPDS, Edinburgh Postnatal Depression Scale; FCI, Family Care Indicators; MSPSS, Multidimensional Scale of Perceived Social Support; SES, socioeconomic status.

Values are β (95% CI).

^1^Adjusted for child sex, mother’s age, mother’s educational attainment (completed secondary education or above, yes vs*.* no), and household socioeconomic status score.

*p ≤ 0.05; **p ≤ 0.01; ***p < 0.001.

Associations between maternal capabilities for care and FCI subscale scores were also explored with mixed findings between countries (Table 5). In Malawi, support with childcare (p < 0.001) and higher levels of perceived social support (p = 0.03) were associated with fewer sources of play materials. Maternal employment (p = 0.05) and higher levels of perceived social support (p = 0.02) were associated with fewer play activities with caregivers in the previous 3 days. Support with childcare (p < 0.001) and higher decision-making (p = 0.03) were associated with greater varieties of play materials. Conversely, higher maternal haemoglobin concentration was associated with less variety of play materials (p = 0.05). However, this association was not significant when a binary variable for anaemia (haemoglobin <120 g/L yes vs. no) was used.

In South Africa, support with childcare was consistently associated with greater sources of play materials (p = 0.003), more varieties of play materials (p = 0.01) and more play activities with caregivers in the previous 3 days (p = 0.05). However, greater decision-making was associated with fewer sources of play materials (p < 0.001) and fewer play activities with caregivers in the previous 3 days (p = 0.02).

## Discussion

In this study, we examined associations between multiple maternal capabilities for care and nurturing care behaviours among mother-child dyads in Malawi and South Africa. Maternal employment and decision-making as measures of autonomy, support with childcare as a proxy measure of reasonable workload and reported social support were the maternal capabilities most consistently associated with different nurturing care behaviours, although the direction of associations differed between contexts. Maternal nutritional status, assessed by haemoglobin concentration, and mental health were associated with one care behaviour each. Associations were sometimes in unexpected directions and although statistically significant, associations were weak, suggesting that multiple factors likely influence nurturing care behaviours.

Maternal employment and decision-making, as measures of maternal autonomy, were largely negatively associated with nurturing care behaviours, including continued breastfeeding, complete child immunisations and stimulation practices. While autonomy and employment may improve health, nutrition and financial status and decision-making in favour of children, it may also negatively impact caregiving. The need to return to paid employment is identified by women as one of the main reasons for early cessation of exclusive and continued breastfeeding [36]. Family support, flexible work schedules and workplace breastfeeding policies have been identified as strategies to support women in work with breastfeeding [37,38]. The negative association of maternal employment with complete child immunisation status among Malawian dyads in the present study contrasts with a pooled analysis of Demographic and Health Survey (DHS) data from 17 sub-Saharan African countries [39]. While employed mothers may have better access to income, transport and healthcare facilities [39], they may not have time to travel to and wait at health clinics for child immunisations. The type of employment (e.g., formal vs. informal) may further influence caregiving. Informal work is characterised by job insecurity, lack of social protection and lower pay, while strategies and structural support for working mothers often focus on formal workplaces [40].

Consistent with our findings, previous research has also demonstrated mixed associations between decision-making and nurturing care behaviours. No associations were found between decision-making autonomy and IYCF indicators or child immunisation status in Zimbabwe [10] and Uganda [11], however positive associations between decision-making autonomy and IYCF indicators, immunisation status and stimulation practices were found in Bangladesh, Ethiopia and Vietnam [9]. It is important to note that maternal decision-making autonomy is just one dimension of women’s empowerment. Analysis of DHS data from nine sub-Saharan African countries found different dimensions of women’s empowerment, including ownership and control of financial and economic resources, decision-making power and gender attitudes, to be differentially associated with child dietary diversity, growth, cognitive development and stimulation activities [41]. Importantly, there is general agreement that women’s empowerment is context-specific and measuring and comparing empowerment across contexts is challenging [42]. DHS indicators of women’s empowerment, such as the questions regarding decision-making utilised in this study, measure aspects of women’s empowerment that are similar across diverse contexts [23] and therefore may not capture contextual aspects of women’s empowerment. Comprehensive measures of universal and context-specific aspects of women’s empowerment are needed.

Mothers in employment as well as those out of employment are typically engaged in time-intensive domestic activities including household maintenance and care of household members leading to time poverty [1]. Adequate and appropriate support with childcare (e.g., with feeding or engaging children in stimulating activities) is therefore important for all mothers [43]. Evidence from sub-Saharan Africa suggests that a higher proportion of 3–4 year old children receive stimulation from other caregivers (19%) than from their mothers (15%) or fathers (4%) [44], in agreement with our findings of positive associations between support with childcare and psychosocial stimulation in both contexts. However, individuals within a mother’s support network may have differing beliefs and knowledge regarding child feeding practices or the importance of providing young children with responsive stimulation and early learning activities [45,46]. As demonstrated by the negative associations between support with childcare and IYCF indicators in the South African cohort in the present study, interventions should effectively engage family members and other caregivers beyond mothers in building support for nurturing care in ways that fit the cultural context [47,48].

Perceived social support was positively associated with MDD among South African mother-child dyads, in agreement with evidence from Ethiopia, Vietnam, Zimbabwe and Uganda [9–11]. However, associations between perceived social support and stimulation practices is mixed, with negative associations among Malawian dyads in the present study, positive associations among dyads in Bangladesh and Vietnam [9], while no association was found in a Kenyan study [49]. Most measures assess general social support, with few considering individual social support dimensions (emotional, informational and instrumental) or social support focused on specific care behaviours. Behaviour-specific social support measures have stronger associations with health behaviours and outcomes compared with general social support measures [50].

Maternal mental health and nutritional status assessed by haemoglobin concentration did not show consistent associations with nurturing care behaviours in this study and the observed significant associations were weak and in unexpected directions. We assessed multiple maternal capabilities and nurturing care behaviours raising the possibility of type I errors. Given the limited literature and the exploratory nature of this study, we did not adjust for multiple comparisons. Previous research has not found any association between poor maternal mental health [49,51] or maternal anaemia [31] and maternal engagement in psychosocial stimulation. Good maternal physical and mental health and adequate maternal nutrition are recognised as important maternal capabilities for nurturing care [1,2] and future research should explore how maternal health and different measures of nutritional status (e.g., maternal undernutrition, dietary intakes) may influence nurturing care behaviours [52].

In agreement with previous studies [9–11], associations between maternal capabilities for care and care behaviours differed between study settings. There may be two potential reasons for these inconsistencies across different contexts. First, care behaviours are influenced by many factors other than maternal or household/family characteristics. Mother’s caregiving practices are situated within larger family and social structures and thus contextual factors such as culture, community beliefs and social norms shape caregiving behaviours, as well as broader national-level economic, political and environmental contexts (Fig 1). Second, a major challenge in interpreting the literature and comparing studies is the wide variety of definitions and tools used to measure different maternal capabilities for care (recently reviewed in detail, [8]). Some constructs lack standardised measures that can be applied in cross-cultural contexts with limited reporting of how these constructs are conceptualised or operationalised in different contexts, while others rely on proxy measures for constructs often available in large-scale datasets. However, taken together, the available evidence suggests a wide range of maternal capabilities for care are important for caregiving and an improvement in the status of mothers overall may translate into better care for young children.

This study has several strengths. First, we included several constructs of maternal capabilities for care. Most research to date has considered individual capabilities for care [8]. Reliable questionnaires validated in each setting were used (e.g., EPDS, MSPSS). However, this study is not without limitations that must be considered in the interpretation of the findings. This secondary data analysis used maternal capability for care constructs, or proxies for these constructs, that were available within the Khula Study. Therefore, unmeasured capabilities for care, such as maternal knowledge or beliefs, physical health and mothering self-efficacy, may play a role in shaping care behaviours. Additionally, while we adjusted for child-, maternal- and household-level variables, maternal capabilities for care may also be influenced by the father’s knowledge or beliefs or social norms in each community. Due to the secondary objectives of the present work, the sample size was not based on the objectives of this secondary data analysis. The cross-sectional nature of the analysis precludes the establishment of causal relationships. Subsequent longitudinal studies may help elucidate temporal associations between maternal capabilities for care, nurturing care behaviours and child growth and development. While our findings shed light on the maternal capabilities for care associated with nurturing care behaviours in urban Blantyre district and peri-urban Gugulethu, our findings are not generalisable to rural-dwelling populations in either Malawi or South Africa or populations in other resource-limited contexts. Finally, maternal capabilities for care and nurturing care behaviours relied on self-reported measures and are therefore subject to social desirability and recall bias (e.g., mothers asked to recall play activities children engaged in with alternate caregivers.

## Conclusions

In summary, this study has helped to identify certain maternal capabilities for care in specific contexts in Malawi and South Africa that are associated with key childcare behaviours, although some associations were weak, suggesting that multiple factors likely influence nurturing care behaviours. However, this contributes to an important and growing body of research and these findings, taken together with previous evidence, highlight that different maternal capabilities for care are integral for different care behaviours encompassed within the Nurturing Care Framework and this varies by context. Interventions promoting nurturing care, which largely require maternal behaviour change to adopt recommended practices, are unlikely to sustainably improve nurturing care without fully understanding the resources and capabilities that mothers and other caregivers in different contexts need. Therefore, having measures of multiple maternal capabilities for care, rather than focusing on individual capabilities, which has often been the case, is crucial. Contextually appropriate interventions should simultaneously reinforce maternal autonomy, social support, nutritional status and mental wellbeing of caregivers, empowering them to enhance their child’s nutrition, growth and development through optimal nurturing care. This should be implemented within the context of enabling family, community and environmental systems. Future research should explore maternal capabilities for care across different contexts using standardised tools, identify causal and possibly underutilised pathways between capabilities and nurturing care behaviours and develop and evaluate interventions that enhance maternal capabilities to nurture children who thrive.

## Supporting information

S1 Checklist Inclusivity in global research. (DOCX)

S1 Fig Flow chart of study participants. (PDF)

S1 Table Definition, categorisation and timing of data collection for each of the maternal capabilities for care indicators. (DOCX)

S2 Table Definition, categorisation and timing of data collection for each of the nurturing care behaviour indicators. (DOCX)

S3 Table Definition, categorisation and timing of data collection for each of the covariate variables. (DOCX)

S4 Table Family Care Indicator (FCI) subscales in Malawi and South Africa. (DOCX)
