# Supplementary material for: Associations between maternal capabilities for care and nurturing care behaviours among mother-child dyads in Malawi and South Africa
Source: PLOS Glob Public Health. 2025 Sep 2;5(9):e0005017. doi: 10.1371/journal.pgph.0005017 (PMC12404457; doi:10.1371/journal.pgph.0005017)
Supplement: S1 Fig — (PDF) [file pgph.0005017.s002.pdf]

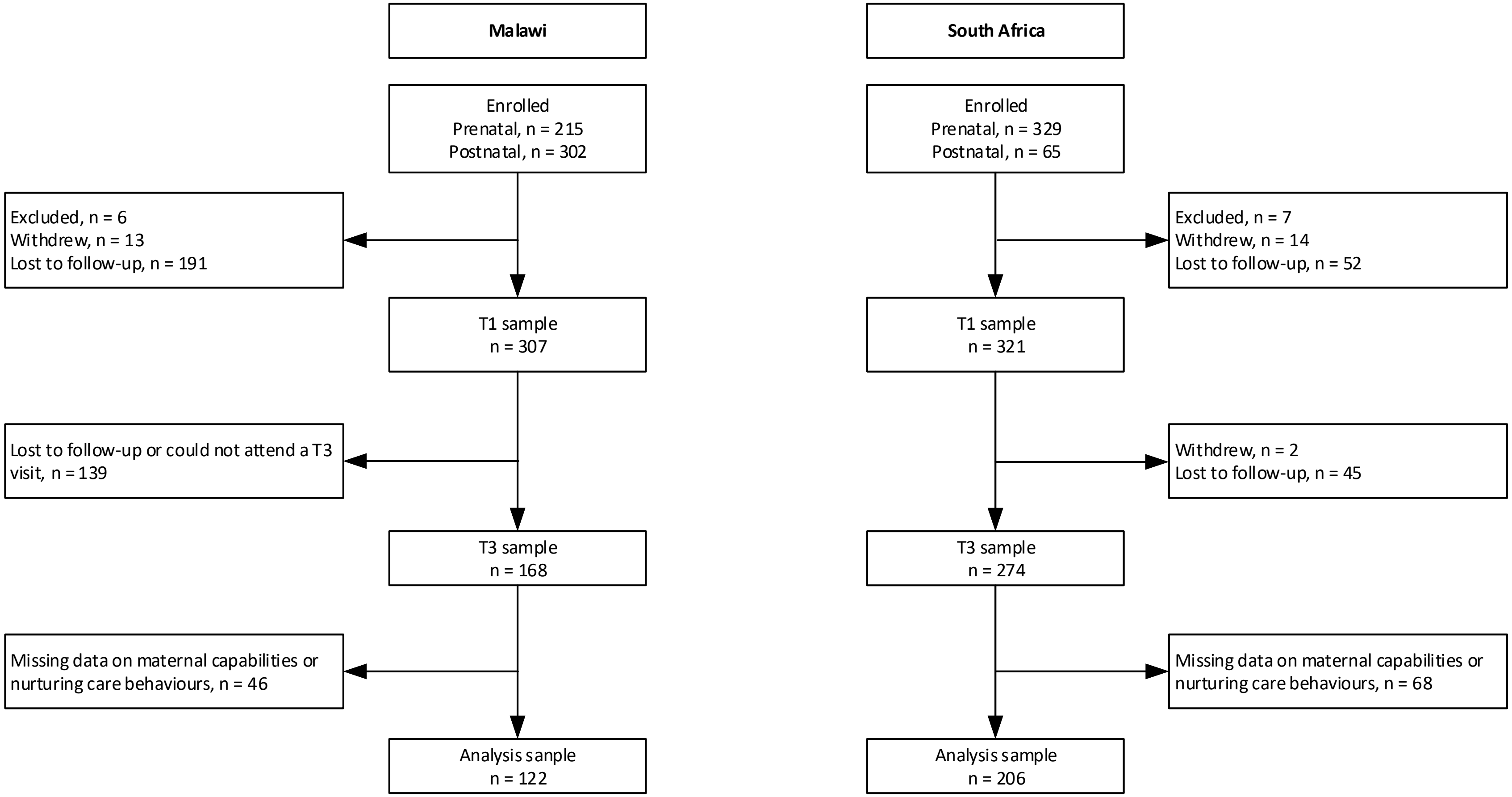

S1 Fig. Flow chart of study participants

T1, first study visit when children were 2 - 5 months of age; T3, third study visit when children were 10 - 16 months of age.

Adapted from Zieff MR, Miles M, Mbale E, Eastman E, Ginnell L, Williams SCR, et al. Characterizing developing executive functions in the first 1000 days in South Africa and Malawi: The Khula Study. Wellcome Open Research. 2024;9(157).
