# Supplementary material for: Associations between maternal capabilities for care and nurturing care behaviours among mother-child dyads in Malawi and South Africa
Source: PLOS Glob Public Health. 2025 Sep 2;5(9):e0005017. doi: 10.1371/journal.pgph.0005017 (PMC12404457; doi:10.1371/journal.pgph.0005017)
Supplement: S1 Table — (DOCX) [file pgph.0005017.s003.docx]

**S1 Table. Definition, categorisation and timing of data collection for each of the maternal capabilities for care indicators**

| **Maternal capabilities for care** | **Definition** | **Variable type** | **Category/score** | **Timing of data collection** |
| --- | --- | --- | --- | --- |
| **Health and nutritional status** | | | | |
| Haemoglobin | Haemoglobin concentration (venous blood assessed by haematology analyser). Adjusted for altitude in Malawi only. | Continuous | N/A | T1 |
| **Mental wellbeing** | | | | |
| Edinburgh Postnatal Depression Scale (EPDS) | Number and frequency of depressive symptoms reported by mothers over the previous 7 days. Higher score indicates a higher number and frequency of depressive symptoms.  Proportion of mothers with EPDS score ≥9. | Continuous  Binary | Score 0 – 30  0 = no; 1 = yes | T3 |
| **Autonomy** | | | | |
| Employed | Proportion of mothers who reported they were employed. | Binary | 0 = no (housewife; unemployed; student)  1 = yes (farmer; office work; vendor) | EN |
| Decision making | Number of household decisions mothers were involved in either alone or jointly with a spouse.   - Household decisions: i) how mothers’ earnings are spent (Malawi only); ii) how partners earnings are spent; iii) mothers health care; iv) major household purchases; v) visiting family and friends. | Continuous | Score 0 – 5 (Malawi)  Score 0 – 4 (South Africa) | T3 |
| **Reasonable workload** | | | | |
| Support with childcare | Proportion of mothers who reported receiving support with childcare from any other caregiver.   - Other caregivers: i) partner; ii) grandparent; iii) sibling; iv) aunt; v) uncle; vi) nanny/childminder. | Binary | 0 = no; 1 = yes | T3 |
| **Social support** | | | | |
| Multidimensional Scale of Perceived Social Support (MSPSS) total score | Mothers’ perception of social support received from a significant other, family and friends. Higher score indicates greater perceived social support. | Continuous | Score 0 – 48 | T3 |

EN, enrolment; T1, first study visit when children were 2 – 5 months of age; T3, third study visit when children were 10 – 16 months of age.
