# Supplementary material for: Associations between maternal capabilities for care and nurturing care behaviours among mother-child dyads in Malawi and South Africa
Source: PLOS Glob Public Health. 2025 Sep 2;5(9):e0005017. doi: 10.1371/journal.pgph.0005017 (PMC12404457; doi:10.1371/journal.pgph.0005017)
Supplement: S2 Table — (DOCX) [file pgph.0005017.s004.docx]

**S2 Table. Definition, categorisation and timing of data collection for each of the nurturing care behaviour indicators**

| **Maternal capabilities for care** | **Definition** | **Variable type** | **Category/score** | **Timing of data collection** |
| --- | --- | --- | --- | --- |
| **Infant and young child feeding** | | | | |
| Continued breastfeeding | Proportion of children receiving breastmilk at around 1 year of age. | Binary | 0 = no; 1 = yes | T3 |
| Minimum dietary diversity (MDD) | Proportion of children who received food from ≥5 out of eight food groups in the previous 24 hours.   - Food groups: i) breastmilk; ii) grains, roots and tubers; iii) pulses, nuts and seeds; iv) milk and dairy products; v) meat, poultry and fish; vi) eggs; vii) vitamin A rich fruit and vegetables; viii) other fruit and vegetables. | Binary | 0 = no; 1 = yes | T3 |
| Minimum meal frequency (MMF) | Proportion of children receiving solid, semi-solid or soft foods at least the minimum number of times in the previous 24 hours.   - 3 times per day for children receiving breastmilk; - 4 times per day for children not receiving breastmilk . | Binary | 0 = no; 1 = yes | T3 |
| Minimum acceptable diet (MAD) | Proportion of children who consumed both the MDD and MMF in the previous 24 hours. | Binary | 0 = no; 1 = yes | T3 |
| **Health-seeking practices** | | | | |
| Full immunisation | Proportion of children who had received all vaccines appropriate for their age.   - Vaccine schedule: birth; 6 weeks; 10 weeks; 14 weeks; 6 months; 9 months; 1 year. | Binary | 0 = no; 1 = yes | T3 |
| **Psychosocial stimulation** | | | | |
| Family Care Indicators (FCI) total score | Summed score of all four subscale scores (presence of books; sources of play materials, varieties of play materials, play activities). Higher score indicates more stimulation and caregiver interaction with the child. | Continuous | Score 0 – 17 | T3 |
| Availability of children’s books | Proportion of caregivers reporting ≥1 children’s book in the home. | Binary | 0 = no; 1 = yes | T3 |
| Sources of play materials | Number of different sources play things available in the home.   - Sources of play materials: i) homemade toys; ii) shop bought toys; iii) household objects. | Continuous | Score 0 – 3 | T3 |
| Varieties of play materials | Number of different types of play things available in the home.   - Varieties of play materials: i) things/toys that play or make music; ii) things for drawing or writing; iii) picture books for children; iv) things meant for stacking/constructing/building; v) things for moving around; vi) toys for learning shapes and colours; vii) things for pretending. | Continuous | Score 0 – 7 | T3 |
| Play activities | Number of activities any household member (≥15 years of age) participated in with the child in the home in the previous 3 days.   - Play activities: i) read books or looked at picture book with child; ii) told stories to child; iii) sang songs to child; iv) took child outside the home; v) played with child; vi) counted or drew things with child. | Continuous | Score 0 – 6 | T3 |

T3, third study visit when children were 10 – 16 months of age.
