# Supplementary material for: Associations between maternal capabilities for care and nurturing care behaviours among mother-child dyads in Malawi and South Africa
Source: PLOS Glob Public Health. 2025 Sep 2;5(9):e0005017. doi: 10.1371/journal.pgph.0005017 (PMC12404457; doi:10.1371/journal.pgph.0005017)
Supplement: S3 Table — (DOCX) [file pgph.0005017.s005.docx]

**S3 Table. Definition, categorisation and timing of data collection for each of the covariate variables**

| **Covariates** | **Definition** | **Variable type** | **Category/score** | **Timing of data collection** |
| --- | --- | --- | --- | --- |
| **Child level** | | | | |
| Child sex | Sex of the child. | Binary | 0 = female; 1 = male | T1 |
| **Maternal level** | | | | |
| Mothers age | Mothers age at enrolment. | Continuous | N/A | EN |
| Mother education | Maternal education defined as either mother having completed secondary level education or above or not. | Binary | 0 = mother has not completed secondary level education or above (none; started primary; completed primary; started secondary)  1 = mother has completed secondary level education or above (completed secondary; some education beyond secondary; university) | EN |
| **Household level** | | | | |
| Household socioeconomic status (SES) | Number of indicators available in the household. A score of 1 was assigned if the indicator was available and 0 if not. Scores were summed for a total SES score, with higher scores indicating a greater SES.   - Indicators: i) electricity; ii) improved water source (piped, public tap, tubewell, protected dug well, protected spring, rainwater); iii) improved sanitation (flush, ventilated pit latrine, pit with slab); iv) man-made flooring (Malawi) or improved dwelling (South Africa; house, flat); v) improved cooking fuel (electricity/electric stove); ownership of ≥1 household asset (Malawi: radio, TV, landline/mobile phone, computer, fridge, freezer, bicycle, scooter, car; South Africa: phone, car/truck, motorcycle/scooter, bicycle, domestic servant). | Continuous | Score 0 – 6 | EN |

EN, enrolment; T1, first study visit when children were 2 – 5 months of age.
