# Supplementary material for: Associations between maternal capabilities for care and nurturing care behaviours among mother-child dyads in Malawi and South Africa
Source: PLOS Glob Public Health. 2025 Sep 2;5(9):e0005017. doi: 10.1371/journal.pgph.0005017 (PMC12404457; doi:10.1371/journal.pgph.0005017)
Supplement: S4 Table — (DOCX) [file pgph.0005017.s006.docx]

**S4 Table. Family Care Indicator (FCI) subscales in Malawi and South Africa**

| **FCI subscale** | **Frequency (%)** | |
| --- | --- | --- |
|  | **Malawi**  **(n = 122)** | **South Africa**  **(n = 206)** |
| **Household books** |  |  |
| None | 86 (70.5) | 145 (70.4) |
| 1-2 | 29 (23.8) | 36 (17.5) |
| ≥3 | 7 (5.5) | 25 (12.1) |
| **Sources of play materials** |  |  |
| Homemade toys | 33 (27.9) | 87 (42.2) |
| Shop bought toys | 74 (60.7) | 167 (81.1) |
| Household objects | 33 (27.0) | 163 (79.1) |
| **Varieties of play materials** |  |  |
| Things/toys that play or make music | 17 (13.9) | 139 (67.5) |
| Things for drawing or writing | 1 (0.8) | 75 (36.4) |
| Picture books for children | 5 (4.1) | 58 (28.2) |
| Things meant for stacking, constructing, building | 2 (1.6) | 57 (27.7) |
| Things for moving around (e.g. balls, tricycles) | 24 (19.7) | 152 (73.8) |
| Toys for learning shapes and colours | 4 (3.3) | 59 (28.6) |
| Things for pretending | 73 (59.8) | 102 (49.5) |
| **Family interaction (play activities in the previous 3 days)** |  |  |
| Read books or looked at picture books with child | 30 (24.6) | 43 (20.9) |
| Told stories to child | 104 (85.2) | 61 (29.6) |
| Sang songs to child | 118 (96.7) | 170 (82.5) |
| Took child outside the home | 120 (98.4) | 160 (77.7) |
| Played with child | 121 (99.2) | 179 (86.9) |
| Counted or drew things with child | 24 (19.7) | 85 (41.3) |
